# Connecting Neurobiological Features with Interregional Dysconnectivity in Social-Cognitive Impairments of Schizophrenia

**DOI:** 10.3390/ijms24097680

**Published:** 2023-04-22

**Authors:** Florian W. Adraoui, Linda Douw, Gerard J. M. Martens, Dorien A. Maas

**Affiliations:** 1 Biotrial, Preclinical Pharmacology Department, 7-9 rue Jean-Louis Bertrand, 35000 Rennes, France; 2 Anatomy and Neurosciences, Amsterdam UMC Location Vrije Universiteit Amsterdam, Boelelaan, 1081 HZ Amsterdam, The Netherlands; 3 Donders Centre for Neuroscience (DCN), Department of Molecular Animal Physiology, Faculty of Science, Donders Institute for Brain, Cognition and Behavior, Radboud University, 6525 GA Nijmegen, The Netherlands; 4 NeuroDrug Research Ltd., 6525 ED Nijmegen, The Netherlands

**Keywords:** schizophrenia, social cognition, functional connectivity/dysconnectivity, structural connectivity/dysconnectivity, oxidative stress, inflammation, N-methyl-D-aspartate receptor

## Abstract

Schizophrenia (SZ) is a devastating psychiatric disorder affecting about 1% of the world’s population. Social-cognitive impairments in SZ prevent positive social interactions and lead to progressive social withdrawal. The neurobiological underpinnings of social-cognitive symptoms remain poorly understood, which hinders the development of novel treatments. At the whole-brain level, an abnormal activation of social brain regions and interregional dysconnectivity within social-cognitive brain networks have been identified as major contributors to these symptoms. At the cellular and subcellular levels, an interplay between oxidative stress, neuroinflammation and N-methyl-D-aspartate receptor hypofunction is thought to underly SZ pathology. However, it is not clear how these molecular processes are linked with interregional dysconnectivity in the genesis of social-cognitive symptoms. Here, we aim to bridge the gap between macroscale (connectivity analyses) and microscale (molecular and cellular mechanistic) knowledge by proposing impaired myelination and the disinhibition of local microcircuits as possible causative biological pathways leading to dysconnectivity and abnormal activity of the social brain. Furthermore, we recommend electroencephalography as a promising translational technique that can foster pre-clinical drug development and discuss attractive drug targets for the treatment of social-cognitive symptoms in SZ.

## 1. Introduction

Schizophrenia (SZ) is a devastating neuropsychiatric disorder affecting around 1% of the world’s population [1]. SZ patients have a complex phenotype that can be divided into positive (e.g., delusions and hallucinations), negative (e.g., anhedonia and reduced motivation) and cognitive (e.g., planning and concentration problems) symptoms [1]. Social-cognitive deficits, the focus of this review and part of negative symptoms, include affected mental processes underlying the perception of, interpretation of, and response to social stimuli [2]. These impairments result in an inability to adapt one’s behavior to match the social context, leading to negative social interactions, reduced social functioning and progressive social withdrawal [2]. Deficits in social cognition start in the prodromal phase of SZ before the onset of the first psychotic episode [3,4,5]. These symptoms not only affect SZ patients and their caregivers but also contribute significantly to the economic burden of SZ [6]. Yet, the neurobiological processes underlying social-cognitive impairments in SZ remain largely unknown.

Social cognition is governed by several brain structures forming the ‘social brain’ [7]. The social brain includes the amygdala (AMY), prefrontal cortex (PFC), orbitofrontal cortex (OFC) and the anterior cingulate cortex (ACC), which serve as substrates for social processing through a number of social-cognitive networks [8]. For instance, the perception network detects social stimuli, relying on the AMY, PFC and the sensory system. In SZ patients, abnormal brain activity and connectivity within the social brain have been observed [2,8,9]. Additionally, in both SZ patients and animal models of SZ, molecular and cellular abnormalities (e.g., oxidative stress, inflammation and N-methyl-D-aspartate (NMDA) receptor hypofunction) have been reported in brain structures that play a key role in the social brain network such as the PFC, ACC and AMY [10,11,12,13,14]. However, the manner in which microscale molecular and cellular abnormalities lead to macroscale brain activity and connectivity deficits within the social brain remains unknown. This leaves a knowledge gap that together with the limited application of translational research methods complicates treatment development [15,16,17,18]. Indeed, the treatment of social–cognitive symptoms of SZ continues to represent an unmet medical need.

In this review, we first describe macroscale scientific knowledge by detailing which brain structures and neural networks contribute to social-cognitive dysfunction in SZ. Next, we discuss the link that exists between key microscale neurobiological factors and impaired social-cognitive behavior in SZ. Importantly, we then connect the current macro- and microscale knowledge about social-cognitive symptoms of SZ and propose that impaired myelination of white-matter tracts and disinhibition of local microcircuits might lead to dysconnectivity within as well as abnormal activity of the social brain, ultimately causing the social-cognitive symptoms of SZ. Finally, we discuss potential drug targets and identify promising translational research techniques that could facilitate the development of medicines for the treatment of social-cognitive dysfunction in SZ.

## 2. Macroscale Knowledge—Brain Structures and Networks That Regulate Social Cognition Are Affected in SZ

### 2.1. Impairments in the Recruitment of the Social Brain during Social-Cognitive Behavior in SZ

Neuroimaging and neuropsychological studies suggest that more than fifteen brain regions contribute to social-cognitive deficits in SZ, including the PFC, OFC, AMY, ACC, superior temporal gyrus and occipital cortex [2,9]. Specifically, increased PFC activation and abnormal PFC control of the AMY are associated with defects in emotion regulation, which complicates social interactions [19,20]. In turn, hypoactivation of the superior temporal sulcus is linked to defects in motor resonance, further hindering social interactions by preventing patients to match to other people’s behavior [21]. Moreover, decreased activation of the medial PFC and OFC is thought to contribute to impairments during theory of mind tasks, likely leading to the inability to infer the mental states of others, which is a process essential for proper social behavior [22,23,24]. Furthermore, the sensory processing of faces and voices is affected in SZ patients, and research suggest this might be associated with a decreased activation of the AMY, ACC, PFC and occipital cortex (for face perception) [25,26,27,28] and abnormal activation of the superior temporal gyrus and insula (for voice perception) [29,30], hindering social interaction. On top of that, attributional style defects in SZ, for instance mistakenly perceived hostility, correlate with a decreased activation of the primary motor cortex, middle cingulate cortex and AMY [31]. What causes these numerous structures within the social brain to malfunction in the process of initiating and maintaining normal social behavior remains unclear, but abnormal connectivity between regions of the social brain might play a role [2,9].

### 2.2. Reduced Structural Connectivity within the Social Brain Affects Social Cognition in SZ

In addition to differences in the activation of brain regions involved in social cognition, evidence points to abnormal structural connectivity within the social brain of SZ patients. Integration of the activity of brain regions in the social brain depends on the transmission of neural information from one region to another via bundles of white matter (WM). WM within the social brain is therefore an important determinant of social-cognitive behavior (see references [32,33] for reviews on major WM tracts involved in social cognition), and abnormal structural connectivity is a key feature of SZ [34,35,36]. Diffusion magnetic resonance imaging (MRI) studies reveal abnormal frontal WM in SZ patients (e.g., in the PFC) [37] that is independent of medication use [38,39], which is evident already in the prodromal phase of SZ and advances to caudal brain regions as the disease progresses [39,40,41,42,43,44]. The integrity of WM in the PFC, a key region for social-cognitive behavior, is decreased in SZ patients [45,46,47], which is associated with poorer socio-functional outcomes [48,49]. Similar associations were found in studies investigating other regions of the social brain. For instance, reduced sociability has been associated with reduced WM integrity within and between the OFC, and anterior and posterior cingulate cortices [50,51,52]. In addition, decreased integrity of the longitudinal fasciculus, a WM bundle supporting theory of mind and perception of social stimuli, predicts the deterioration of social functioning in adolescents who are at a high risk to transition to psychosis [53], highlighting the importance of WM tracts for the emergence of social deficits in SZ. Indeed, reduced WM integrity in the corpus callosum, occipital cortex and anterior corona radiata of SZ patients, supporting theory of mind, mirroring and perception networks, is correlated with impaired social functioning [54,55,56]. Moreover, abnormal integrity of the inferior fronto-occipital fasciculus, whose fibers contribute to face perception and mentalizing, is correlated with social deficits in 22q11.2 deletion syndrome, which is a genetic condition conferring increased risk for SZ [57]. Likewise, decreased integrity of the cingulum, which allows connection within the mentalizing network, and the longitudinal fasciculus have both been associated with lower performance in the theory of mind task in SZ patients [58]. Additionally, reduced integrity of the uncinate fasciculus, a bundle of WM connecting the AMY to the OFC and PFC, and important for the regulation of emotions, was correlated with decreased ability to process emotions in SZ patients [59,60,61]. Taken together, these studies support the idea that reduced WM integrity throughout the social brain is a major contributor to the incorrect activation of and communication between regions of the social brain, resulting in social-cognitive deficits in SZ patients. Yet, the quality of the functional connections within social brain networks may also play a role in generating social-cognitive symptoms in SZ.

### 2.3. Abnormal Properties of Functional Social Brain Networks in SZ

Functional connectivity studies employ functional MRI, electroencephalography (EEG) or magnetoencephalography (MEG) to establish brain activation patterns. Subsequently, statistical associations between activation patterns in distinct regions of the brain are determined, revealing a matrix containing all pairwise connectivity values between brain regions. Functional network analysis of the social brain in SZ patients might therefore provide us with more insight into how differential functional connectivity and reduced structural connectivity of social brain regions might integrate to disrupt social cognition in SZ. Social brain networks in SZ patients are characterized by a lower functional connectivity between social brain regions, and the functional connections between social brain regions are often not direct but go via more other brain regions than in healthy individuals. This indicates less efficient communication within the social brain of SZ patients [62]. Notably, AMY and putamen are less centrally connected within the social brain network of SZ patients, and this was correlated with social-cognitive deficits in SZ [62]. When looking at the theory of mind brain network specifically, the PFC was found to be the major disconnected brain region, indicating that the PFC likely receives less input from other brain regions within the theory of mind network in SZ patients. Lower connectivity within the theory of mind network in SZ patients correlated with worse interpersonal behavior [63,64]. Interestingly, in healthy individuals, a correlation exists between the functional social brain network and the real-life social network (i.e., the number of social interactions and social connections someone maintains), and this correlation is reduced in SZ patients [64], which is a notion that was confirmed in a second study [65]. Functional network analysis of the social brain in SZ patients thus tells us that particularly, the regions of the social brain are less efficiently connected. Abnormalities in WM connecting social brain regions in SZ patients are therefore of interest in the search for novel treatment strategies for social-cognitive impairment in SZ.

In conclusion, aberrant activity and connectivity throughout the social brain of SZ patients causes social-cognitive deficits. Social-cognitive training, aiming at improving social cognition in SZ patients, has been shown to slightly improve specific domains of social cognition such as emotion recognition [66,67]. However, social-cognitive training comes with a high burden of treatment. The development of medication or therapy that directly targets the dysconnectivity within the social brain of SZ patients depends on the identification of biological treatment targets. As such, it is important to investigate the molecular and cellular features that lead to changes in the development and functioning of connections within the SZ social brain.

## 3. Microscale Knowledge—Molecular and Cellular Mechanisms Underlying Social-Cognitive Dysfunction in SZ

It is thought that a combination of genetic and environmental factors leads to a series of pathological processes including oxidative stress, neuroinflammation and NMDA receptor hypofunction that disrupt brain development and ultimately cause SZ [10,11,68]. These pathological processes are interconnected, aggravate one another [68] and affect neurotransmitter systems, the activation of brain regions, and interregional brain connectivity [10,11]. Therefore, it is likely that these mechanisms contribute to the abnormal connectivity within the social brain, leading to social-cognitive dysfunction in SZ. In this section, we describe the link between social-cognitive dysfunction in SZ and oxidative stress, immune irregularities and a decrease in NMDA receptor signaling.

### 3.1. Oxidative Stress Is Associated with Social-Cognitive Impairments in SZ

Oxidative stress is an imbalance between the production and the clearance of reactive oxygen species (ROS). ROS can damage cells and cause cell death [69], but they are also essential for cellular processes such as immune functions [70]. Therefore, maintaining a balance between the production and clearance of ROS is essential for proper physiological functioning. In SZ, oxidative stress is thought to result from a combination of mitochondrial dysfunction producing elevated ROS levels [71,72,73,74,75] and decreased capacity for clearance of ROS due to lower glutathione antioxidant levels [76,77,78,79,80]. This may result from both genetic factors such as single nucleotide polymorphisms (SNPs) and copy number variations involved in genes responsible for maintaining the redox balance [76,77,78], and environmental insults such as maternal immune activation (MIA), prenatal malnutrition and social stress, which all increase ROS production [81,82,83]. Oxidative stress is a key feature of SZ, and it is observed throughout the brain (including the social-brain regions PFC, occipital cortex and ACC) [84,85,86,87], the blood [88] and the cerebral spinal fluid [84] of patients. Moreover, oxidative stress is already present in the prodromal phase of SZ and is therefore thought to importantly contribute to the disorder (see references [89,90] for reviews). Social-cognitive deficits in SZ have been correlated with oxidative stress. For instance, lower plasma total antioxidant status was associated with poorer emotional management in SZ patients [91], and a magnetic resonance spectroscopy study revealed a correlation between lower glutathione levels in the frontal cortex and severity of social dysfunction in SZ patients [92,93]. In agreement, it was found that in SZ patients, serum redox imbalance was strongly associated with social withdrawal [94]. However, other studies measuring specific components of the redox system, e.g., super oxide dismutase, did not find associations with social-cognitive measures [95,96], suggesting that the total oxidative balance rather than the dysregulation of specific components of the redox system dictates association with social-cognitive deficits in SZ. In line with this, N-acetylcysteine (NAC), a direct precursor of the brain’s main antioxidant glutathione, ameliorates indices of social functioning in SZ [97,98,99,100], indicating a causal link between oxidative stress and social dysfunction. In addition to social improvement, NAC treatment also reversed pathological electrophysiological brain features associated with social cognition deficits. For example, mismatch negativity (MMN), an EEG paradigm in which a specific electrophysiological pattern is triggered upon detecting a deviant stimulus within a sequence of standard cues and a measure associated with SZ patient’s impaired social cognition [101,102,103,104], was improved in SZ patients undergoing NAC treatment [105]. These studies suggest that oxidative stress contributes to social-cognitive dysfunction in SZ and that rescuing oxidative stress might improve social cognition in SZ patients.

Studies in various rodent models of SZ strengthen the notion that oxidative stress affects social cognition in SZ. For instance, impairment of the glutathione pathway induced by L-buthionine-(S, R)-sulfoximine reproduces key aspects of SZ in rats including impaired social behavior [106,107], which is rescued by NAC administration [108]. Furthermore, rats socially isolated from birth onwards, a model for studying SZ since post-natal psychosocial stress is a risk factor for SZ [109,110], have higher levels of oxidative stress in the frontal cortex, which was associated with decreased social interactions [111]. In addition, in rodents treated with the NMDA receptor antagonists ketamine, phencyclidine or MK-801 (a well-characterized model of SZ; see reference [112]), oxidative stress is induced in the brain and accompanied by deficits in social behavior [113,114,115] that are ameliorated by NAC [116,117]. NAC treatment also reverses oxidative stress and social interaction deficits (as well as other behavioral manifestations relevant to SZ) induced by MIA and methamphetamine exposure during adolescence in rats [118], which is a relevant neurodevelopmental model of SZ since both adolescent drug exposure and MIA are known to contribute to SZ susceptibility [119,120,121,122]. Similarly, oxidative stress in the PFC of rats exhibiting social deficits induced by perinatal infection and adolescent psychological stress is rescued by NAC treatment [123]. Interestingly, oxidative stress has also been found in the AMY in a model of MIA and is reduced by the administration of minocycline (7-dimethylamino-6-dimethyl-6-deoxytetracycline), which is an anti-inflammatory antibiotic also displaying antioxidant properties [124]. Evidence from rodent studies is thus in line with clinical findings suggesting a link between oxidative stress in the social brain and social-cognitive impairments in SZ.

### 3.2. Immune Dysregulation Is Associated with Social-Cognitive Impairments in SZ

Another main pathological process in SZ is immune dysregulation and neuroinflammation. Inflammation occurs when immune cells (i.e., white blood cells or microglia) become activated upon the identification of potential bodily threats. The activation of immune cells leads them to secrete pro-inflammatory cytokines that regulate the immune response. Immune dysregulation and neuroinflammation are key components of SZ pathophysiology [125]. SZ patients exhibit abnormal levels of pro-inflammatory cytokines in the blood, cerebral–spinal fluid and brain (including the PFC) [126,127,128], microglia are abnormally activated in SZ patients’ brains [129,130,131], and genetic and genome-wide association studies have identified major histocompatibility complex genes involved in inflammatory processes as major contributors to SZ genetic susceptibility [132,133,134,135]. In addition, early-life exposure to environmental stressors such as MIA or social stress induces microglial activation and neuroimmune dysregulation, contributing to the development of SZ [129,130,131]. Like oxidative stress, heightened inflammatory processes are observed from the prodromal phase of SZ onwards, suggesting a major contribution of immune dysregulation to the development of SZ [129,130,131].

Social behavior strongly depends on inflammatory status [136,137], and it has even been suggested that impaired social interactions prevent the exposure to bacteria necessary for a proper development of the immune system, highlighting the interdependence of social-cognitive behavior and the immune system [138]. Therefore, a contribution of immune dysregulation to social-cognitive impairments in SZ patients is to be expected. Indeed, higher plasma levels of inflammatory components such as interleukin 10 (IL-10) predict social-functioning impairments [139]. Another study even found that higher IL-10 levels lead to misinterpretation of social cues and that elevated IL-2 correlates with other social-cognitive measures in SZ patients [140,141]. Likewise, plasma levels of interferon (IFN)-γ, IL-1β and IL-12 negatively correlate with indices of social cognition (e.g., theory of mind) in SZ patients [142]. In addition, the anti-inflammatory antibiotic minocycline benefits social functioning in SZ patients [143,144,145,146,147,148], which is an effect thought to be mediated by its action on pro-inflammatory cytokines [148]. Taken together, these studies point to an association between immune dysregulation and social-cognitive symptoms in SZ patients.

A causal link between immune dysregulation and social deficits appears to exist in SZ rodent models as well. Immune activation models are among the most commonly used SZ rodent models and associated with social deficits. For instance, in a rat juvenile immune activation model of SZ, deficits in social recognition and interactions have been found [149], and MIA in rats induces communication and social interaction deficits [150]. Notably, MIA SZ models induce neuroinflammation and microglia activation in regions of the social brain including AMY and PFC [124,151]. Several other studies suggest that prenatal and early-life inflammation are key to inducing social deficits in SZ [152,153,154], but we should note that NMDA-antagonism models also present with neuroinflammation in the social brain (e.g., in the PFC), which is a component that may also contribute to the social deficits observed in this model (see reference [113] and paragraph below). This body of evidence prompts the idea that prenatal and early-life inflammation might impact brain development of the social brain, notably in the AMY and PFC, leading to social deficits later in life.

### 3.3. NMDA Receptor Hypofunction Is Associated with Social-Cognitive Impairments in SZ

Next to oxidative stress and neuroinflammation, NMDA receptor hypofunction is considered a key contributor to the development of SZ based on the fact that the administration of NMDA receptor antagonists in healthy individuals induces psychosis-like states [155] and that SNPs in glutamate-associated genes carry genetic susceptibility for SZ [132,156]. Additionally, post-mortem brain tissue from SZ patients displays reduced levels of NMDA receptors (see reference [157] for review) and SZ animal models induced by NMDA receptor antagonists cause SZ-like behaviors including decreased cognitive performance and sensory processing as well as persistent social deficits [112,158].

In patients with SZ, low ACC glutamate levels correlate with decreased social functioning [159], and thalamus glutamate levels show a negative correlation with social functioning [160]. To our knowledge, these are the only studies that have investigated a possible correlation between glutamatergic signaling and social cognition in SZ patients. Yet, post-mortem studies have identified a reduced expression of NMDA receptors in multiple areas of the social brain in SZ patients, including the ACC and PFC [161,162,163], strengthening the notion that NMDA receptor hypofunction may contribute to social-cognitive impairments in SZ. Furthermore, auditory steady-state response (ASSR) and MMN, two EEG event-related potentials that are dependent on glutamatergic activity, are reduced in SZ patients [104,164] and predictive of SZ socio-functional deficits [101,102,103,165,166,167,168,169,170,171,172,173], highlighting a possible link between glutamatergic hypofunction and social cognition in SZ. In line with this, studies in SZ animal models demonstrate a clear association between glutamatergic hypofunction and social deficits. SZ rodent models induced by NMDA receptor antagonism present with deficits in social interactions [112,158], as do rodents in which NMDA receptor subunit genes are knocked down [174,175]. Interestingly, in NMDA-receptor-antagonism-induced SZ models, aberrant activity of glutamatergic fibers projecting from the AMY to the ACC has been recently shown to contribute to this deficit [176]. Furthermore, in SZ patients and animal models, drugs stimulating glutamatergic transmission through metabotropic receptors (e.g., mGluR2/3) or by increasing the synaptic concentration of the NMDA receptor co-agonists glycine or D-serine improve symptoms of SZ including deficits in social interactions [177,178,179,180,181,182,183,184,185], thus establishing that NMDA receptor hypofunction is a significant factor contributing to social deficits in SZ.

Taken together, oxidative stress, neuroinflammation and NMDA receptor hypofunction are considered the main pathological processes in the development of SZ, and all three processes contribute to social-cognitive impairments in SZ patients and rodent models. However, it remains unclear how these microscale mechanistic contributors can cause dysconnectivity within the social brain of SZ patients. In the next section, we will discuss how the three molecular and cellular mechanisms might contribute to the whole-brain abnormalities that cause social-cognitive disturbances in SZ.

## 4. Connecting the Macro- and Microscales in SZ Social-Cognitive Research

As indicated by the neuroimaging and network studies highlighted above, there appears to be a reduced structural connectivity as well as functional dysconnectivity within the social brain in SZ patients, and this may impact social cognition. Since oxidative stress, immune dysregulation, neuroinflammation and NMDA receptor hypofunction are also associated with social-cognitive deficits in SZ, exploring how these microscale factors impact local cortical circuit output as well as interregional connectivity may shed light on the underpinnings of SZ social deficits.

### 4.1. The Effects of Oxidative Stress, Neuroinflammation and NMDA Receptor Hypofunction on Local Brain Circuits

Oxidative stress can affect all brain cell types, but parvalbumin interneurons (PVIs) and oligodendrocytes are particularly vulnerable to this type of cellular stress. Notably, PVIs, their integration in local neural circuits (e.g., in the PFC, hippocampus or occipital cortex) and their interaction with oligodendrocytes are essential for proper social-cognitive processes and could contribute to the development of social deficits in SZ [186,187,188,189,190]. PVIs are fast-spiking cells with a correspondingly high metabolic rate and high numbers of mitochondria, thus producing more ROS than other interneuron types and their excitatory counterparts [191]. Therefore, PVIs are more vulnerable to oxidative insults than other neuronal cell types. Indeed, in SZ, post-mortem PFC tissue PV and GAD67 mRNA and protein expression are reduced [192,193,194,195,196], while PVI numbers remain unchanged [197,198] as do synapse numbers [199,200]. Furthermore, in the PFC of an SZ rat model, increased oxidative stress leads to reduced glutamic acid decarboxylase 67 (GAD67) mRNA and protein expression but unchanged numbers of γ-Aminobutyric acid (GABA)ergic interneurons [201,202,203]. Reduced PV and GAD67 expression indicates a lower activity of these interneurons in the SZ PFC, which has been confirmed in SZ rodent models [203]. A lower activity of fast-spiking interneurons leads to a disinhibition of the local circuit and a reduction of cortical gamma-band oscillations that has been observed both in SZ patients and in oxidative stress-related animal models of SZ [204,205,206]. Strengthening the notion that oxidative stress affects PVIs is the fact that in both rat and mouse models of SZ with diminished antioxidative capacity, decreased numbers of PVIs have been identified [204,207]. Importantly, disturbing the excitation/inhibition balance by modulating PVI functioning or the specific knock-down of PVIs in the PFC has been shown to disrupt social behavior in laboratory animals [208,209].

In addition to detrimental effects on interneurons, oxidative stress also heavily affects oligodendrocytes. Oligodendrocytes are glial cells that form myelin sheaths around neuronal axons, which not only enhances conduction velocity of action potentials but also provides metabolic support to axons. Myelin is an extension of the oligodendrocyte cell membrane, and one oligodendrocyte can myelinate up to 40–50 axons. This action comes with a high metabolic rate, and high lipid and protein production rates, which are processes that produce ROS and make oligodendrocytes vulnerable to oxidative insults [10,11]. Considering the role of oxidative stress in SZ, it is therefore not surprising that myelination abnormalities represent also an important feature of this disorder [210,211,212,213,214,215], which is a circumstance mainly mediated during brain development and notably affecting the PFC [10,11]. In a recent series of publications, we indeed demonstrated that in a rat model of SZ, oxidative stress during brain development impairs oligodendrocyte maturation and leads to a reduced PVI myelination rate in the PFC [201,202]. Interestingly, it has been proposed that the hypomyelination of PVIs may further contribute to the reduced PVI functionality observed in SZ [216]. Taken together, oxidative stress contributes to a disinhibition of local cortical circuits through detrimental effects on PVIs and oligodendrocytes, which might affect the activation of the social brain and social behavior in patients.

The exact effects of neuroinflammation on local cortical circuits in SZ remain largely unknown. However, insights from the MIA model of SZ suggest effects on both interneurons and dopaminergic neurons. The MIA model of SZ causes a decreased number of PVI in the frontal cortex [150] and decreased forebrain and hippocampal expression of genes involved in PVI development [217,218]. Reduced PVI transmission due to lower release probability was confirmed by another rodent MIA study in which the impaired PVI functionality was shown to lead to abnormalities in gamma band oscillations [219], while the deficits in social behavior displayed by this model seem to depend on incorrect GABAergic-mediated ACC function [220]. Moreover, in the hippocampus of the MIA SZ model, reduced GAD67 protein expression per interneuron but no changes in interneuron numbers have been observed and were accompanied by a lower coherence in all EEG frequencies between PFC and hippocampus, indicating that local intraneuronal changes might impact interregional activity in regions important for social cognition [221]. The effect of neuroinflammation on interneurons was further shown in a study in which activated microglia caused long-lasting metabolic changes in interneurons derived from induced pluripotent stem cells from SZ patients that led to decreased mitochondrial function and reduced arborization [222]. These studies highlight that, like oxidative stress, neuroinflammation might lead to reduced interneuron functioning in forebrain circuits and likely beyond to subcortical areas. Interestingly, an interplay between neuroinflammation and dopamine has also been suggested. For example, in an MIA SZ model, reduced numbers and firing rates of ventral tegmental area dopamine neurons were reported [223]. Another MIA study confirmed reduced firing rates of vental tegmental area dopamine neurons and additionally reported increased baseline dopamine levels in the nucleus accumbens but not the PFC [224]. The interplay between neuroinflammation and dopamine is further highlighted by the fact that dopamine signaling through the dopamine D1 receptor downregulates inflammasome activity [225]. These last studies make the link with the dopaminergic hypothesis of SZ indicating lower mesocortical and higher mesolimbic dopaminergic activity [226] and suggest that neuroinflammation could cause a dopaminergic imbalance further contributing to improper local neural network functioning. Therefore, neuroinflammation has detrimental effects on interneurons in local cortical circuits and on dopaminergic transmission, which might influence regional neural activity and consequently social behaviors.

In addition to oxidative stress and neuroinflammation, NMDA receptor hypofunction in SZ also has effects on local neural circuitry. It is thought that NMDA receptor hypofunction mainly affects cortical interneurons, resulting in a lower excitation rate [227]. All types of cortical interneurons express NMDA receptors, which confers on them a central role in social cognition as they determine the activity of local pyramidal neurons that project to other parts of the brain [228]. NMDA receptor hypofunction in (PV) interneurons leads to a disinhibition of cortical pyramidal neurons, increasing the output of cortical regions and potentially inducing glutamate spillover from synapses, which could lead to spine degeneration [229]. Indeed, in SZ, a decreased expression of synaptic genes in the post-mortem frontal cortex and hippocampus has been reported [230]. Similar findings suggest the PFC from animal models of SZ involving NMDA receptor hypofunction exhibits decreased synapse numbers [231,232] as well as an excitation/inhibition imbalance [233].

Taken together, oxidative stress, neuroinflammation and NMDA receptor hypofunction all contribute to abnormalities in PVIs that lead to a disinhibition of local cortical circuits and potentially to abnormal neural activity in brain regions of crucial importance for social behavior. In the long term, local disinhibition could lead to excitotoxicity, damaging neural cells and connections between brain regions and ultimately causing the dysconnectivity that is observed in SZ neuroimaging studies.

### 4.2. Oxidative Stress, Neuroinflammation and NMDA Receptor Hypofunction Might Impact Interregional Connectivity through WM Damage within the Social Brain

Having established that oxidative stress, neuroinflammation and NMDA receptor hypofunction affect local cortical circuits, we next wondered whether any direct and indirect effects of these pathological processes could impair connections between regions of the social brain. As discussed above, a disconnection within the social brain of SZ patients has consistently been observed, and the connections between brain regions depend largely on WM bundles. These bundles contain myelinated and unmyelinated axons as well as glial cells. Redox imbalance causes myelination deficits in the PFC of a rat SZ model [202] as well as decreased structural integrity of the anterior commissure and fornix WM in mice [234], suggesting that oxidative stress has a direct effect on myelination and WM tracts. In line with this, NAC antioxidant treatment can ameliorate myelin abnormalities not only in a rat model of SZ associated with redox imbalance [202] but also in demyelination mouse models induced by cuprizone [235] that exhibit SZ-like features including reduced social interactions [236,237,238,239]. Interestingly, NAC also reversed social deficit in these demyelination models, further highlighting a possible contribution of oxidative stress-induced myelin defects to SZ social deficits [235]. In fact, in SZ patients, NAC treatment also benefits neural connections by increasing functional connectivity within the cingulate cortex [240] and by increasing the structural connectivity of the fornix [241], suggesting that restoring redox imbalance might benefit myelin and WM bundles important for social cognition in SZ.

Inflammatory markers have also been associated with WM quality measures, notably that of corpus callosum, both in SZ patients and controls [242]. In SZ, the levels of the pro-inflammatory cytokine IL-6 were correlated with lower integrity of the genus of the corpus callosum and the anterior limb of the internal capsule [243]. Similarly, increased levels of IL-10 pro-inflammatory cytokines were also associated with the disruption of WM integrity of, amongst other regions, the corpus callosum in SZ patients [244]. IL-6 and C-reactive protein inflammatory markers also correlated with WM integrity in the inferior longitudinal fasciculus and the inferior fronto-occipital fasciculus in SZ patients but not controls [245]. As for oxidative stress, neuroinflammation could cause WM deficiency via its effect on oligodendrocytes that are particularly susceptible to inflammatory processes [246]. In line with this idea, altered WM is recapitulated in MIA rodent models in which disruptions in WM integrity within the social brain were found to arise from oligodendrocyte changes (e.g., reduced expression of myelin-related enzyme 2′,3′-cyclic nucleotide 3′-phosphodiesterase) [247]. Other studies on the MIA SZ model confirmed the occurrence of lower mRNA expression levels of myelin- and oligodendrocyte-related genes [248] and reduced myelination [249]. Furthermore, a systematic review concluded that microglial activation is associated with SZ in white- rather than gray-matter brain areas [250], while activated microglia containing myelin debris were found in SZ WM alongside apoptotic oligodendrocytes [12,250]. It therefore seems likely that oxidative stress and neuroinflammation damage oligodendrocytes and myelin in WM bundles connecting social brain regions in SZ. The demyelination observed in WM between (among others) the PFC [12] and the cingulum from SZ patients further strengthens this notion [251]. In addition, corpus callosum proteomics studies found a dysregulation of proteins involved in myelination as well as energy metabolism in SZ patients [252]. Interregional connectivity within the social brain through WM bundles might therefore be directly affected by oxidative stress and neuroinflammation.

NMDA receptor hypofunction may also play a role in mediating hypomyelination in the social brain of SZ patients. For instance, in rodent studies, MK-801 exposure was found to induce demyelination, decrease WM volume as well as the expression of myelin and oligodendrocyte markers, induce myelin sheath degeneration in the corpus callosum [253,254] and decrease myelin-related gene expression in the AMY [255]. This leads to the speculation that the disinhibition of local cortical circuits caused by oxidative stress, neuroinflammation as well as NMDA receptor hypoactivation may lead to an increased glutamatergic activity of axons in WM bundles connecting social brain regions. In line with this idea, increased glutamate release by axons might exert excitotoxic effects on oligodendrocytes [256], thereby damaging myelination and leading to dysconnectivity in the long term.

**Figure 1:**
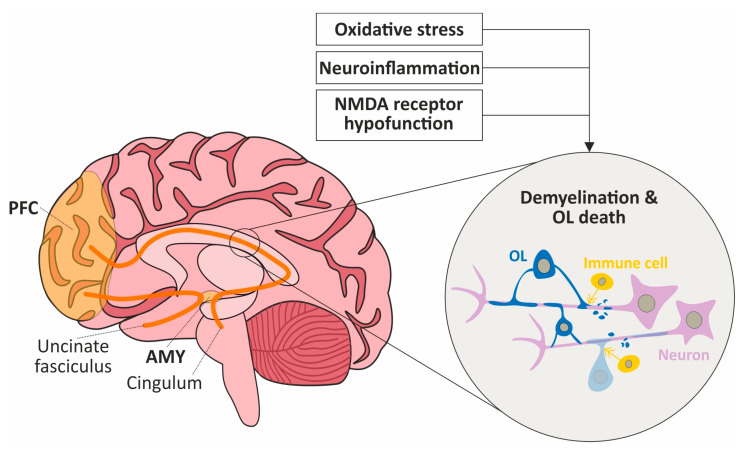
Schematic representation of the deleterious effects of neurobiological processes involved in schizophrenia on WM tracts supporting social-cognitive networks; example of altered PFC-AMY connectivity. Oxidative stress, neuroinflammation and NMDA receptor hypofunction may damage WM by reducing myelination and inducing oligodendrocyte (OL) death, for instance at the level of the uncinate fasciculus and cingulum that both allow connections between the social-brain regions PFC and AMY. This mechanism may also occur within social brain regions (e.g., PFC) and at the whole social-brain level and may consequently impact social cognition and associated behaviors.

In summary, microscale pathological processes may have both direct and indirect detrimental effects on the myelin of WM bundles connecting brain regions and cause the disinhibition of local circuits. Figure 1 illustrates how such pathological processes could damage WM connecting the main regions of the social brain such as the AMY and PFC. This highlights the need for a pre-clinical investigation of both macro- and microscale brain networks instead of singular brain regions in the disruption of social cognition in SZ.

## 5. Implications for Translational Research and Drug Development

### 5.1. Promising Pharmacological Candidates for the Treatment of Social-Cognitive Impairments in SZ

In view of the above, drug developers could consider molecules impacting key pillars of SZ pathophysiology to target social-cognitive deficits. Among the most-promising candidates are first and foremost drugs targeting oxidative stress such as NAC that displays beneficial effects on key aspects of SZ (e.g., connectivity) and several symptoms of SZ patients including emotional management and social deficits [97,98,99,100,240]. In addition, pharmacological modulators of glutamatergic transmission should be considered. For example, metabotropic glutamate receptor modulators and compounds modulating the synaptic concentration of the NMDA receptor co-agonists glycine and D-serine show promising pre-clinical and clinical outcomes in SZ [177,178,179,180,181,182,183,184,185]. Aiming at restoring a normal inhibitory control of local microcircuits could also be of interest for drug developers trying to treat SZ social dysfunction. For instance, the use of positive allosteric GABA receptor modulators recovers social deficits as well as other symptoms in animal models of the disorder [257,258]. Other compounds such as serotonin type-3 receptor blockers may be of interest because of their potential to ameliorate the excitation/inhibition imbalance though actions on interneurons and have already shown positive effects on the MMN response in SZ [259]. Molecules rescuing immune dysregulations and neuroinflammation may be considered as well. One example concerns the anti-inflammatory antibiotic minocycline, whose benefits for social functioning in SZ patients have been described [143,144,145,146,147,148]. As there seems to be an important interplay between oxidative stress, neuroinflammation and the local disinhibition of neural circuits, drug developers could also aim at targeting multiple aspects of the pathophysiology at the same time. This could be the case for drugs similar to pregnenolone, a neurosteroid and anti-inflammatory compound that also modulates the excitation/inhibition imbalance [260] and significantly decreases negative symptoms of SZ patients [260,261]. As network analysis studies suggest that disconnection within the social brain is a key determinant affecting social cognition in SZ patients, novel strategies may involve the improvement of WM integrity impairments by stimulating the survival of oligodendrocytes and production of myelin, which is a strategy notably employed in drug development for multiple sclerosis whose pathophysiology shares several similarities with SZ (e.g., inflammation and myelination deficits as well as cognitive symptomatology; see reference [262] for a review). However, to the best of our knowledge, there are no reports yet on the possible amelioration of social deficits in SZ by drugs acting on myelination. It is nonetheless worth noting that a number of pathways involved in oligodendrocyte proliferation and differentiation, including one that contains the mammalian target of rapamycin, have been proposed as attractive targets because of their potential roles in oligodendrocyte malfunctioning in SZ [11,263]. Therefore, focusing on such molecular pathways may lead to positive outcomes in SZ drug development. Drug development studies may also benefit from improved translational approaches, which is discussed next.

### 5.2. Better Translational Methods Could Improve Drug Development for Social-Cognitive Impairments in SZ: EEG as an Example

Despite the promising drug candidates described above, there is still no effective pharmacological treatment available that ameliorates the social-cognitive symptoms of SZ. This is partly due to the fact that drugs effective in pre-clinical settings often lack efficacy in clinical trials, which is an outcome that is probably due to the limited translation of output parameters from pre-clinical to clinical investigations [15,16,17,18]. Indeed, the leading strategy in pre-clinical drug development research over the past decades has been to evaluate drug candidates targeting SZ symptoms by assessing rodent behavior with limited translational potential and without clear neurobiological measures [18]. An additional problem is that most of the behavioral characterizations performed in rodents do not align with the complex panel of ethological responses and their neurological substrates in humans [18,264]. This strategy thus leaves a translational gap in treatment development that is likely responsible for the limited success of SZ drug development. Hence, robust, quantitative and translational methods to characterize disease neurobiology are needed for more prolific drug development. An example of a promising translational technique is EEG. EEG can be used to identify evolutionarily conserved neural activity patterns in both animals and humans. This technique therefore has an exceptional translational value [18,265] and could be used to assess the therapeutic efficacy of investigational drugs on specific neural circuits relevant to SZ pathophysiology. For instance, ASSR and MMN are EEG-recordable neural activity patterns triggered upon the detection of sensory cues by subjects and highly similar in humans and rodents [18,265]. Importantly, ASSR and MMN rely on proper local neural microcircuit functioning and neural pathway integrity and are therefore considered a measure of local neural network functioning. Correct NMDA receptor functioning and inhibitory control exerted by PVIs are indeed crucial in the genesis of ASSR and MMN responses [170,266,267,268,269], which is a process disrupted in SZ and thought to affect social behaviors (see paragraphs above and refs [186,187,208]. In addition, preserved WM integrity appears to be important to elicit a normal MMN response [270]. The MMN and ASSR EEG-evoked potentials are disrupted in SZ [104,164], correlate with social symptoms and are therefore proposed as robust translational windows into impaired social-cognitive processes [101,102,103,165,166,167]. The translational potential of EEG readouts has been confirmed in the context of SZ drug development [164,271], and as a result, molecules targeting key aspects of SZ neurobiology (e.g., NAC addressing oxidative stress or NMDA modulators inducing glutamatergic signaling) generate strong interest from drug developers. Such molecules have shown beneficial effects in patients on both electrophysiological measures and social symptoms [97,105,177,181,182,183,272,273], while a number of traditional antipsychotics such as clozapine or olanzapine (ineffective against social-cognitive symptoms) fail to produce similar EEG effects [274,275,276,277,278], highlighting the need for better translational output measures in drug development. Furthermore, future drug development studies might consider combining EEG with structural and/or functional MRI. Adding MRI outcome measures will further enhance the translational value of such studies, as it will allow assessing both the structure and function of whole-brain networks in both humans and animals [279]. Hence, the implementation of structural and functional MRI as well as EEG-based event-related potential analyses in both pre-clinical and clinical studies represents a promising avenue for the development of medicines targeting social-cognitive deficits in SZ.

## 6. Conclusions

**Figure 2:**
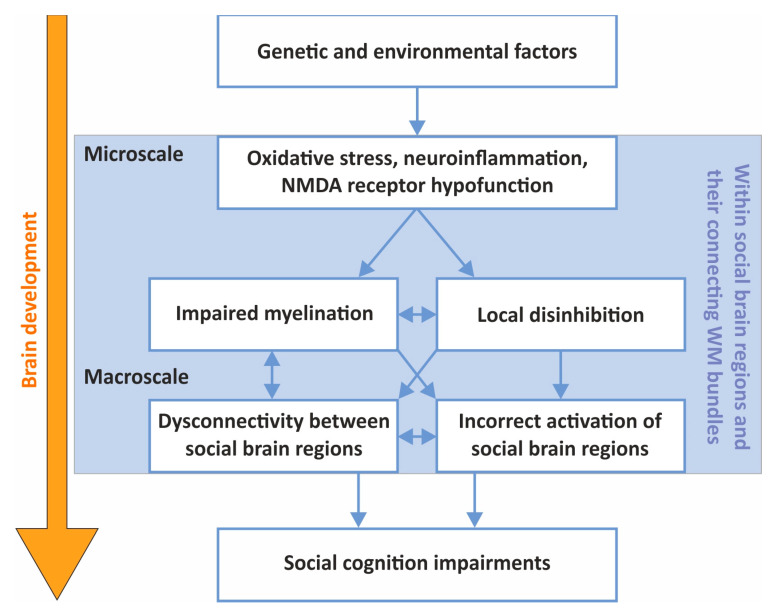
Chart summarizing the proposed link between micro- and macroscale factors involved in the development of social-cognitive symptoms of schizophrenia.

Although further investigations are necessary to fully understand the neurobiological origins of social-cognitive impairments in SZ, current scientific knowledge indicates that oxidative stress, neuroinflammation, NMDA receptor hypofunction, as well as their interplay may contribute. These microscale pathological processes may cause the disinhibition of local neural circuits and have both direct and indirect detrimental effects on myelinated fibers in WM bundles connecting regions of the social brain. This could explain the dysconnectivity that has been observed in the SZ social brain and why social behavior is affected in SZ (Figure 2). As such, there is a need for pre-clinical investigation of local brain microcircuits as well as large-scale neural networks instead of single brain regions with respect to their role in the disruption of social cognition in SZ. We propose EEG as a promising translational measure that together with identified drug targets has the potential to increase the likelihood of success in drug development endeavors to treat social-cognitive deficits of SZ.

## Data Availability

Not applicable.
